# Evaluating the combined effects of ballast water management and trade dynamics on transfers of marine organisms by ships

**DOI:** 10.1371/journal.pone.0172468

**Published:** 2017-03-20

**Authors:** Katharine J. Carney, Mark S. Minton, Kimberly K. Holzer, A. Whitman Miller, Linda D. McCann, Gregory M. Ruiz

**Affiliations:** 1 Marine Invasions Research Laboratory, Smithsonian Environmental Research Center, Edgewater, Maryland, United States of America; 2 Smithsonian Environmental Research Center, Romberg Tiburon Center, Tiburon, California, United States of America; National Sun Yat-sen University, TAIWAN

## Abstract

Global trade by merchant ships is a leading mechanism for the unintentional transfer of marine organisms, including non-indigenous species, to bays and estuaries worldwide. To reduce the likelihood of new invasions, ships are increasingly being required to manage their ballast water (BW) prior to discharge in coastal waters. In the United States, most overseas arrivals have been required to manage BW discharge since 2004, primarily through ballast water exchange (BWE), which flushes out ballast tanks in the open ocean (>200 miles from shore). Studies have found BWE to generally reduce the abundance of organisms, and the amount of water exchanged has been estimated at 96–100%. Despite its widespread use, the overall effect of this management strategy on net propagule supply through time has not been explored. Here, temporal changes in zooplankton concentrations and the volume of BW discharged in Chesapeake Bay, U.S. were evaluated, comparing pre-management era and post-management era time periods. Chesapeake Bay is a large port system that receives extensive BW discharge, especially from bulk cargo vessels (bulkers) that export coal overseas. For bulkers arriving from overseas, mean zooplankton concentrations of total and coastal indicator taxa in BW did not decline between pre- (1993–2000) and post management (2012–2013) eras, when controlling for season and sampling method. Moreover, bulkers discharged 21 million tonnes (82% of total for Chesapeake Bay) of overseas BW in 2013, representing a 374% increase in volume when compared to 2005. The combination of BW discharge volume and zooplankton concentration data indicates that (a) net propagule supply by bulkers has increased since BWE began in Chesapeake Bay; and (b) changes in vessel behaviour and trade have contributed strongly to this outcome. Specifically, the coal-driven increase in BW discharge volume from 2005–2013, concurrent with the onset of BWE regulations, worked to counteract intended results from BW management. A long-term analysis of bulker arrivals (1994–2013) reveals a 20-year minimum in arrival numbers in 2000, just when the implementation of BWE began. This study underscores the need to consider shifts in trade patterns, in order to advance and evaluate effective management strategies for biological invasions.

## Introduction

Global trade has expanded dramatically in recent decades [1], increasing in rate, speed, and geographic extent. This expansion is largely responsible for increases in human-assisted species transfers throughout the world [2–6]. Most trade is conducted by commercial ships, which are known to carry diverse and abundant assemblages of organisms associated with ballast water (BW) and outer hull surfaces [7–13]. Moreover, in coastal ecosystems, transfer of species by ships is a dominant mechanism of invasions by non-indigenous species (NIS), and has driven the observed increases in newly detected invasions over the past century [14–17].

For the past several decades, a stepwise series of management and policy actions have been undertaken to reduce the likelihood of invasions associated with ships’ BW. Early voluntary guidelines have been replaced by mandatory management requirements, which have continued to evolve. For example, BW management (e.g. ballast water exchange (BWE)), has been required by the United States Coast Guard (USCG) since 2004 for most commercial overseas arrivals that intend to discharge BW to U.S. waters; although there are exemptions for vessel safety (e.g. bad weather) and if the vessel does not travel beyond 200 nautical miles (nmi) for sufficient time to conduct BWE [18]. The USCG implemented a concentration-based discharge standard that is being phased in based on vessel class and date of construction [19]. Similar mandatory requirements exist in numerous other nations, and also in the recently ratified IMO Ballast Water Convention [18]. While treatment technologies are at various stages of development and testing, none have been adopted broadly for routine use in the U.S. and elsewhere. Therefore, BWE is still viewed generally as the ‘Best Available Technology’ [as defined in: 20] and for the past decade has remained the primary management method to treat BW discharged into ports [21,22].

As a management method, BWE replaces coastal water with open ocean water (>200 nmi offshore) by emptying and refilling or flushing tanks at sea. This is intended to reduce the concentration of viable coastal organisms delivered to ports and bays, and consequently the probability of establishment for coastal NIS, since the likelihood of establishment is density and frequency dependent [23–25]. Generally, BWE has been shown to decrease the abundance of plankton present in tanks, although diversity has been observed to increase post-exchange [26–33]. While BWE does not replace 100% of water and organisms present in ballast tanks, the amount of water exchanged has been estimated at 96–100%, when experimentally quantified within ballast tanks on commercial ships [24,28,34–36].

Despite the documented efficacy of BWE on a per ship basis, multiple external factors affect the actual concentration of organisms delivered in BW. For instance, various studies since the 1980s have measured the shipboard abundance and diversity of plankton communities, quantifying BW biota at the point of discharge and also their dynamics during voyages [e.g. 13,28,29,37,38]. These studies show high variance in the concentration and composition of plankton observed in BW, independent of BWE or other treatment methods. This variance results from spatial and temporal variation in both (a) the plankton entrained in BW tanks and (b) the effects of specific voyage characteristics (e.g. duration, ocean temperature) and tank characteristics (size, type, and physical and chemical environmental conditions) on survival. For example, total plankton concentrations have been observed frequently to decrease with voyage length [27,28,34,39,40], although an ‘incubator’ effect has also been observed occasionally for some taxa which have increased during a voyage [39]. Moreover, it is possible that plankton concentrations in tanks that underwent BWE could be greater than those that did not, even on vessels travelling the same routes, simply because initial concentrations can vary by orders of magnitude. As a result of such high variation among individual voyages, and the potential for changes in the biotic communities, the net effect of BWE on the propagule pressure (i.e. the number of NIS released to an environment) to a recipient region is uncertain.

This study provides a detailed analysis of historical changes in endpoint zooplankton concentrations present in BW, and annual discharge volumes of BW delivered into Chesapeake Bay, U.S., before and after the implementation of BWE. These data provides a rare synthetic analysis of long-term effects of BWE on net zooplankton delivery to a major port system. Chesapeake Bay was identified as the study location because it is home to two major commercial shipping ports: Baltimore, Maryland and the Port of Norfolk/Newport News (hereafter “Port of Norfolk”), Virginia. The Port of Norfolk is sixth in the ‘Top 10 busiest ports in the U.S.’, its place driven by high volumes of container and bulk cargo trade [41]. In addition to high vessel traffic, Chesapeake Bay also receives a relatively high volume of BW discharge annually, because it is a major overseas export hub for coal by bulk vessels, which discharge BW upon arrival [42,43].

The overall goal of this study was to evaluate long-term changes in BW zooplankton delivery coincident with the transition to, and full implementation of, BWE on ships travelling to Chesapeake Bay. First, concentrations of zooplankton (total and coastal indicator taxa) in overseas BW delivered to Chesapeake Bay in a post-management era sampling period (2012–2013) are compared to those of a pre-management era sampling period (1993–2000), contrasting managed versus unmanaged BW, respectively. Second, following the required implementation of BWE by overseas arrivals to the U.S., changes in overseas BW discharge volume and coal export for Chesapeake Bay are evaluated from 2005–2013. Third, changes in bulk vessel traffic to Chesapeake Bay over a 20-year time period are characterised, from 1994–2013. Finally, the ‘compensatory effects’ of changes in the volume of BW discharge (which offset (counterbalance) the increased use of BWE on propagule supply) and the implications for management, are considered.

## Methods

### Comparison of pre- and post-management

The relative concentrations of zooplankton in BW samples from 1993–2000 (pre-management era) and 2012–2013 (post-management era) were compared. (The zooplankton concentration data are considered ‘relative’ as filtration efficiency is less than 100% [44].) These years correspond to the project’s sampling period, which encompasses BW practices before and after implementation of BWE regulations respectively. Samples were collected and analysed using standardised methods. In each era, BW samples were collected only from bulk cargo vessels (hereafter “bulkers”) arriving to Chesapeake Bay from overseas ports between May and September, corresponding to the season of high zooplankton abundance in coastal source waters [43]. Vessels were boarded and sampled at five coal terminals across Chesapeake Bay: CNX Marine Terminal (Baltimore MD), Chesapeake Bay Piers (Baltimore, MD), Dominion Terminal Associates (Newport News, VA), Kinder Morgan (Newport News, VA), and Norfolk Southern (Norfolk, VA).

For this comparison, the BW from 20 bulk vessels was sampled in the pre-management era and that from 30 bulk vessels was sampled in the post-management era. The north-eastern Atlantic Ocean and Mediterranean Sea were identified as the source regions of BW for >85% of the samples in each era (Table 1). All of the tanks sampled in the pre-management era were reported to have unexchanged BW, while vessels reported BWE occurred for all tanks sampled in the post-management era. For the latter, flow through exchange was the most common method of BWE, reported by 63% of vessels (data not shown). Crew members explained that this preference was due to safety concerns associated with empty-refill exchange.

**Table 1:** Ballast water source and management type for all vessels in pre- and post-management eras. By era shown are: (a) contribution of managed BW for each source region as a percentage of total managed BW, and (b) percentage contribution of BW management methods within each source region. (FT = flow through, ER = empty-refill, None = no management, PU = the ballast tank was pressed up; here water was added to completely fill the tank in mid-voyage). If no vessels of a certain category were sampled this is denoted with a ‘-‘.

Source region	Pre-management			Post-management		
	% total	% BW		% total	% BW	
		None	PU		FT	ER
Asia:						
*South Korea*	3.7	100	0	0	-	-
Eastern Mediterranean:						
*Israel*	37	60	40	0	-	-
*Turkey*	3.7	100	0	0	-	-
Europe:						
*Belgium*	14.8	100	0	3.3	0	100
*Finland*	7.4	50	50	0	-	-
*France*	7.4	50	50	6.7	0	100
*Germany*	0	-	-	10	100	0
*Italy*	0	-	-	10	66.7	33.3
*Netherlands*	7.4	100	0	36.7	72.7	27.3
*Spain*	0	-	-	10	33.3	67.7
*UK*	7.4	100	0	20	83.3	16.7
North America:						
*Panama*	3.7	100	0	0	-	-
*Trinidad*	7.4	50	50	0	-	-
South America:						
*Colombia*	0	-	-	3.3	0	100

In both pre- and post-management eras, the BW samples were collected from a single BW tank per ship, along with transit and BW history. For each tank, the vessel’s BW log was used to characterise the following attributes: port of arrival, last port, BW capacity and BW history. Ballast water history comprised self-reported date, location and volume details for: (1) uptake, (2) management and (3) discharge of ballast water, as well as management method (empty-refill, flow through, pressed up and unexchanged). For BW sampling, all ballast tanks were accessed through manholes with diameters over 30 cm. In each tank, surface and mid-tank (1–12 m range) water temperature and salinity were recorded with a handheld oxygen, conductivity, salinity and temperature system (YSI Model 85). To characterise zooplankton viability, concentration and composition, two replicate vertical tows were conducted using a plankton net (30 cm-diameter conical net, 80 μm mesh size). Net tows were hauled by hand at an approximate rate of 30 cm s^-1^ through the accessible water column (2–24m range), and were measured to the nearest cm. Organisms are heterogeneously stratified within ballast tanks, and thus a net tow may not provide a sample fully representative of the tank but does provide a standardised abundance measure to compare between tanks and vessels.

Zooplankton samples were returned to the laboratory within 30 minutes, or aerated and kept cool if more time was required for transport (30–60 minutes), and then analysed immediately. In the laboratory, zooplankton were examined with a dissecting microscope to assess the proportion of live versus dead organisms. Immobile organisms were considered dead if unresponsive to the physical stimulus of a dissecting probe (i.e. ‘poke test’). The fraction of dead organisms was consistently low across all samples. This was quantified for all samples collected in 2012 by direct counts of the number of dead organisms for the entire sample, or a standardised subsample (using methods described below) when total abundance exceeded 100 individuals.

After initial microscopic examination, zooplankton were preserved in 5% formalin for subsequent enumeration of organisms by taxonomic group. When abundance was low (<100 organisms), the entire sample was enumerated. Most other samples were sub-sampled using a 2 ml-spool Hensen Stempel™ pipette to obtain a representative sample (>100 organisms per sub-sample). For each sample or subsample, individuals were quantified by coarse taxonomic group, such as phylum (e.g., Cnidaria), class (e.g. Bivalvia) or order (e.g. Calanoida) under a binocular dissecting microscope (see S1 Table for taxonomic level information). The number of samples and specimens precluded species-level identifications, particularly for benthic invertebrate larval stages and copepod nauplii, many of which are undescribed. Moreover, the objective was to make comparisons across vessels (BW tanks), which have different species compositions, requiring analysis at a higher taxonomic level.

All 1993–2000 samples were recounted using 2012–2013 methods to ensure consistency. Total recount concentrations showed a strong relationship (r^2^ = 0.9977) to the initial counts, but the recount data were consistently (an average of 25.42%) lower than initial counts. Therefore, recount data presented in this study have been adjusted (increased by 25.42%) to account for this loss. Lower totals in the recount data could be due to multiple factors including: organism degradation during sample storage (≤ 20 years in ethanol), organism loss during counting preparation (i.e. during filtration, rinsing, transfer), and/or potential analyst bias.

Zooplankton concentrations (individuals per m^3^) per sample were calculated for each taxonomic group and total zooplankton by standardising zooplankton abundance based on tow height according to the equation:

$$Zooplankton\;concentration=zooplankton\;abundance_{\;tow}/\left[\pi \;*\;radius^{2}{}_{net}\;*\;height_{\;tow}\right]$$

The major taxonomic groups were further designated and combined into one of two broader groups: (1) ‘coastal indicator taxa’, or (2) ‘other non-indicator taxa’ (see S1 Table). Coastal indicator taxa are those taxonomic groups distinctly associated and largely restricted to bays and nearshore (<50 nmi from shore). Other non-indicator taxa include taxonomic groups that occur in the open ocean but may occur in bays and nearshore communities or are unresolved, such as copepod nauplii. Some of the non-indicator taxa undoubtedly include species with known coastal origin, but this does not apply to the higher-level taxonomic group as a whole. Thus, these taxonomic groups may have mixed (coastal and oceanic) assemblages that are not easily partitioned by habitat preference, especially for larval and juvenile forms. As such, these measures are conservative (minimal) estimates of coastal zooplankton densities, and are intended to provide a robust and standard measure that can be compared directly among samples and time periods.

### Temporal changes in ballast water delivery (2005–2013)

Data from the National Ballast Information Clearinghouse (NBIC) were used to characterise total volume of BW delivered to Chesapeake Bay between 2005 and 2013. The USCG requires nearly all vessels capable of carrying BW to report to the NBIC for each U.S. arrival port, and provide a detailed transit and BW history. The transit history includes arrival, last, and next port of call. For each arrival, vessels report BW source and discharge locations, volumes, and management for any BW carried (whether or not it is scheduled to be discharged) in U.S. waters. Ballast water discharge into Chesapeake Bay was characterised based upon these reports submitted between 2005 and 2013. This period was chosen because reporting compliance (and extent of data) improved dramatically after June 2004, when new BW management requirements (USCG Regulation D-1 [20]) took effect. Between 2005 and 2013, the reporting rate by overseas (i.e. the transit entered the combined US and Canadian Exclusive Economic Zones [EEZ]) and coastwise (i.e. the transit was within the combined U.S. and Canadian EEZ) arrivals to Chesapeake Bay is estimated to be 87.5% [45]. For this nine-year period, changes were measured in the reported annual volume of BW discharge for all vessels and bulk cargo vessels arriving to Chesapeake Bay ports. The relationship between annual BW discharge volume and (a) quantity of coal exported and (b) number of bulk vessel arrivals to Chesapeake Bay was examined. Data for coal exports were obtained from the Energy Information Administration [46].

### Long-term changes in ship arrivals (1994–2013)

Changes in ship arrivals to Chesapeake Bay were estimated over a 20-year period to evaluate potential long-term changes in BW delivery and transfer of associated organisms. A comprehensive picture of ship arrivals required synthesis of multiple data sets covering different time periods, including (a) two Marine Exchanges (MarEx); (b) the Department of Transportation’s Maritime Administration (MARAD); and (c) the USCG’s National Vessel Movement Center (NVMC). The MarEx dataset documents arrivals from 1994–2005 for the two major port complexes in Chesapeake Bay, as reported by the Baltimore Maritime Exchange in Baltimore, Maryland and the Hampton Roads Maritime Exchange in Norfolk, Virginia (hereafter “Maritime Exchange data”). The MARAD dataset consists of “entry records” (arrivals) of vessels from 1994–2005 for the U.S., which are maintained and reported by MARAD as U.S. Foreign Waterborne Transportation Statistics. The NVMC dataset is compiled and maintained by USCG, using required data reports for advanced notice of arrivals, and was available from 2004–2013. While both MarEx and MARAD datasets are also available after 2005, the NVMC dataset is more comprehensive [47] and therefore was used exclusively from 2006–2013.

Using the synthesised dataset, changes were examined in the total number of vessel arrivals and the contribution of bulkers from overseas, as the latter is the major source of BW discharge to Chesapeake Bay [43, see Results]. Based on the arrival and last ports of call in the MARAD and NVMC datasets, each ship arrival was classified as either overseas or coastwise. The MarEx data were used as another estimate of total arrivals. In addition, arrivals in the MARAD and the NVMC data were classified by ship type, to determine the relative contribution of the various ship types to total arrivals and how this changed across the 20-year period.

### Statistical analyses of shipboard sampling data

Zooplankton concentration data were analysed using two sample t-tests, to analyse differences between pre- and post-management era samples in multiple ways. Tests were conducted separately for differences between eras when evaluating: total zooplankton, total zooplankton without copepod nauplii, and coastal indicator taxa only. In the second comparison, copepod nauplii and juveniles were removed from the total zooplankton abundance (due to their high concentration) to determine whether they had masked other patterns in the pre- and post-management era concentration data. In addition, total zooplankton concentrations were compared between: flow through vs. empty-refill BWE methods (post-management era only), and coastal indicator taxa concentration vs. other non-indicator taxa concentration (both eras).

The effects of voyage length (i.e. time since uptake) and BW age (i.e. time since last BW activity (BWE or uptake)) on zooplankton concentration in pre- and post-management eras were analysed using two sample t-tests. The salinity of BW between pre- and post-management eras, and between empty-refill and flow through methods of BWE, was compared using two sample t-tests. All reported values below are shown as mean ± standard error.

Further analyses used linear regressions to test for relationships between (1) ship arrivals (total and bulker) and BW discharge volumes and (2) BW discharge volume (total and bulker) and coal export volumes. These analyses assessed the utility of arrival numbers and coal export volumes in predicting changes in BW discharge volumes in Chesapeake Bay.

## Results

### Temporal changes in zooplankton concentration of ballast water

**Fig 1:**
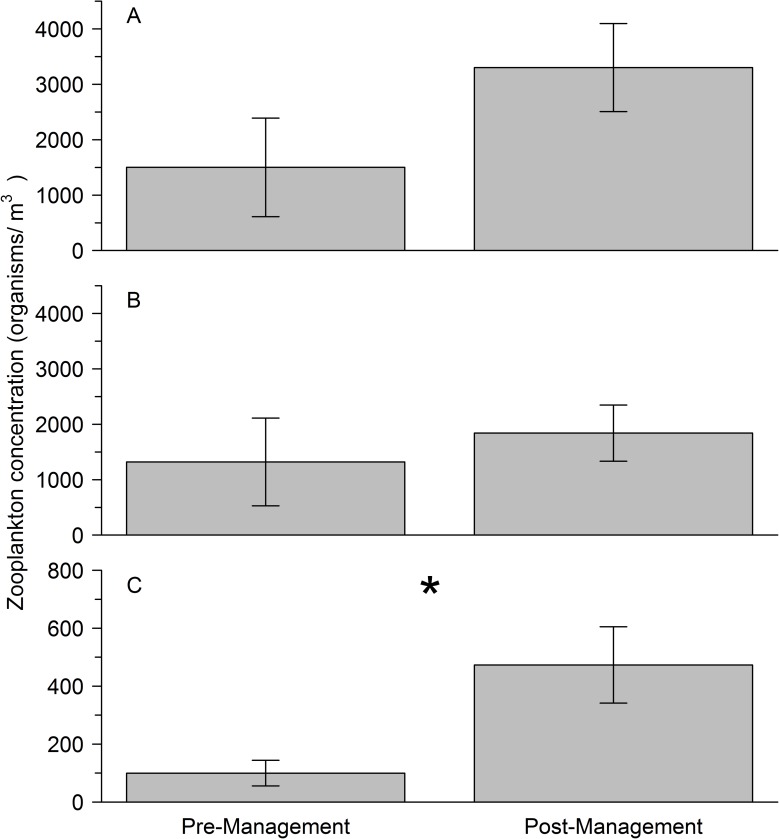
**Concentrations of zooplankton in pre- and post-management era ballast water samples, for (A) total zooplankton, (B) total zooplankton minus copepod nauplii, and (C) coastal zooplankton designated taxa only.** Values are the mean of 20 samples and 30 samples ± SE for pre-management (unexchanged) and post-management (exchanged), respectively. * shows statistically significant data.

The mean total zooplankton concentration was higher in the post-management era, although no significant difference was detected between samples collected from pre-management (unexchanged) and post-management (exchanged) BW (Fig 1A; t-test: t = -1.946, d.f. = 47.39, *p* = 0.058), reflecting high variation among samples. Copepod nauplii and copepodite stages, accounted for 12.1% and 44.3% of mean total abundance in pre-management and post-management data, respectively. Unlike many other taxa present, these larval stages could arise from hatching of eggs (reproduction) during a voyage, obscuring or swamping other patterns (see Discussion). By removing the copepod nauplii and copepodites from the abundance data the pre-management and post-management mean zooplankton concentrations were more similar and still were not significant. (Fig 1B; t-test: t = -0.9454, d.f. = 39.94, *p* = 0.350).

When evaluating coastal indicator taxa alone, there was a significant difference in the mean concentration observed in the pre- and post-management eras (Fig 1C; t-test: t = -2.874, d.f. = 32, *p* = 0.007). The mean concentration was five-fold higher in the post-management BW, despite the reported use of BWE for all tanks. Coastal indicator taxa accounted for 14.3% of mean total zooplankton for samples in the post-management era and 10.4% of those in the pre-management era.

For the post-management samples, zooplankton concentration was higher in tanks exchanged with empty-refill BWE (3,747 ± 1,307 individuals m^-3^) versus flow through BWE (2,932 ± 981 individuals m^-3^), though this difference was not statistically significant (t-test: t = 0.6909, d.f. = 27, *p* = 0.4955). A diverse range of zooplankton was observed in samples: the most abundant groups were the Copepoda (Calanoida, Cyclopoida, Harpacticoida, Poecilostome, copepod nauplii and juvenile stages) and Mollusca (Bivalvia and Gastropoda). The fraction of live zooplankton in samples was consistently high independent of tank history, accounting for an estimated 97 ± 1% of total zooplankton (n = 14 [samples examined live]).

**Fig 2:**
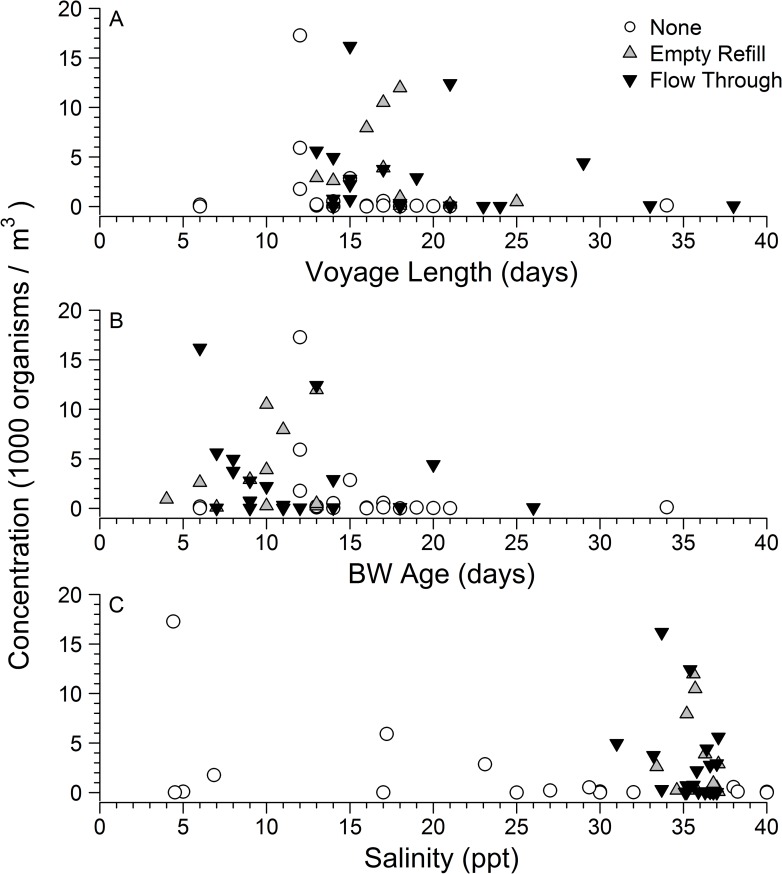
Zooplankton concentration (unexchanged, empty-refill and flow through exchange) with respect to voyage length, ballast water age and salinity. (A) Voyage length (i.e. time from uptake to time of discharge) vs. zooplankton concentration (none, empty-refill and flow through exchange). (B) Ballast water age (i.e. time from BWE to time of discharge) vs. zooplankton concentration (none, empty-refill and flow through exchange). (C) Salinity vs. zooplankton concentration (none, empty-refill and flow through exchange).

In addition to the method of BWE, the biological composition of ballast water will be a function of the location from which it is sourced, and the duration in which it remains in a ballast water tank (see Discussion). Mean voyage length in the pre-management era (15.5 ± 1.4 days) did not differ significantly from that of the post-management era (18.9 ± 1.1 days) (t-test: t = -1.919, d.f. 38.23, *p* = 0.06243). The unmanaged samples showed low concentrations after a voyage length of 12 days, while the total concentration was highly variable in BWE (empty-refill and flow through) samples up to 21 days old (potentially due to a ‘refreshing effect’ of BWE, see Discussion), after which relatively low concentrations were observed (Fig 2). The longer voyage lengths in the pre-management era correspond to vessels from the eastern Mediterranean, including Israel and Turkey, and this was a dominant source (40.7%) of BW that was not represented in the post-management era (Table 1). By removing these data (voyage length > 16 days) from the analyses, higher plankton concentrations were observed in pre-management era data (all data: $\bar{\text{x}}$ = 1501 individuals per m^3^; eastern Mediterranean excluded: $\bar{\text{x}}$ = 2239 individuals per m^3^). Coastal indicator zooplankton concentrations however, were still significantly higher in the post- compared to pre-management era (t-test: t = -2.362, d.f. = 27.65, *p* = 0.0255).

The mean BW age was significantly lower (10.9 ± 0.8 days) in the post-management era compared to the pre-management era (15.5 ± 1.4 days) (t-test: t = 2.887, d.f. = 30.47, *p =* 0.007). Zooplankton concentrations were generally low after 14 days, corresponding mostly to vessels in the pre-management era from the eastern Mediterranean with some tanks that had “pressed up” (added) seawater in the open ocean in the absence of BWE (Table 1 and Fig 2B).

Finally, a significant difference was observed in salinity between pre- and post-management era tanks (t-test: t = -3.347, d.f. = 18.28, *p* = 0.0035) resulting from the absence of low (<31 ppt) salinity water in these tanks that is consistent with expected outcome from BWE (due to the high salinity of water in open ocean BWE locations, see Discussion). Unexchanged vessels in the pre-management era showed lower mean salinity (25.6 ± 3.0 ppt), consistent with uptake in coastal port environments. No significant difference was observed in salinity between tanks that underwent empty-refill (35.9 ± 0.4 ppt) and flow through (35.5 ± 0.3 ppt) BWE (t-test: t = 0.830, d.f. = 26.17, *p* = 0.4139). The total concentration of zooplankton was highly variable, with no relationship to the salinity of the BW, though unmanaged samples showed less variation than BWE samples (Fig 2C).

### Temporal changes in ballast water discharge volume and coal export (2005–2013)

**Fig 3:**
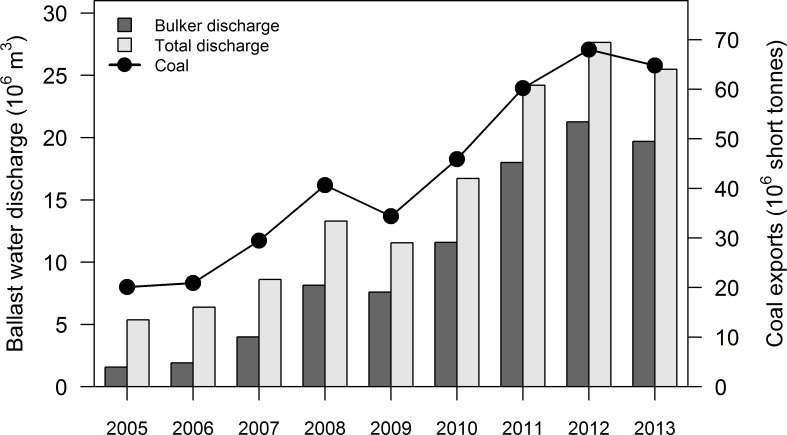
Total volume of ballast water discharged from all vessels types (total) and total overseas (coastal uptake location) ballast water discharged by bulkers arriving at Chesapeake Bay ports annually between 2005 and 2013 [45]. Volume of coal exported from the Ports of Baltimore and Norfolk located in Chesapeake Bay between 2005 and 2013 [46].

**Fig 4:**
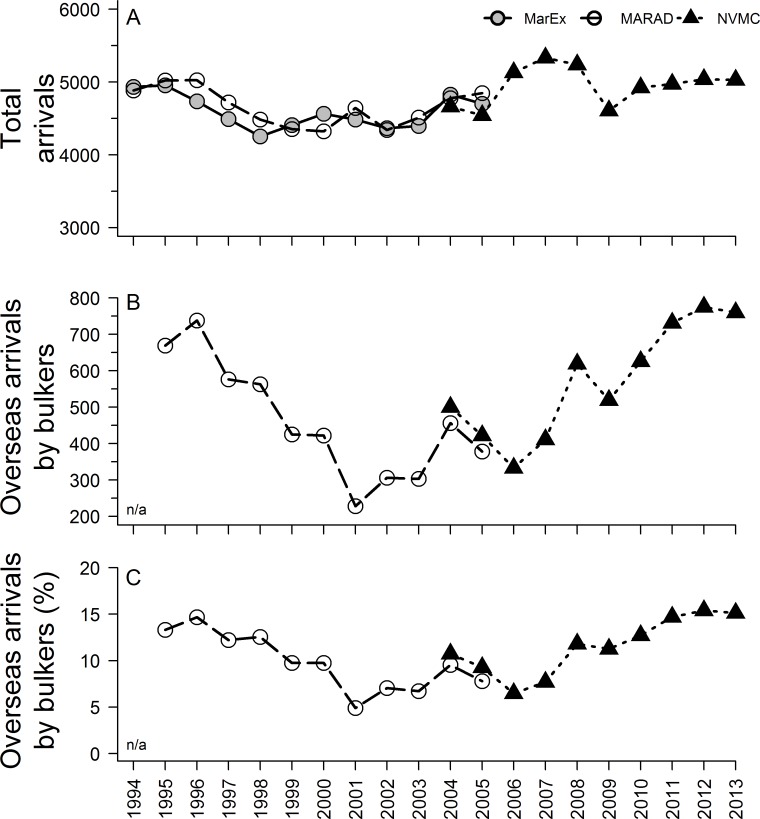
The number of total vessel arrivals and bulker arrivals to Chesapeake Bay between 1994 and 2013. (A) Arrivals to Chesapeake Bay based upon reports to the Baltimore and Hampton Roads Maritime Exchanges (MarEx) and MARAD from 1994–2005 and to NVMC from 2005–2013. (B) The number of overseas arrivals by bulk cargo vessels to Chesapeake Bay ports as reported to MARAD and NVMC. (C) The percentage of all arrivals to Chesapeake Bay that were overseas arrivals by bulkers as reported to MARAD and NVMC. n/a: Vessel type data were incomplete in 1994 and were excluded from analysis.

Total overseas annual BW discharges to Chesapeake Bay increased by 374% (from 5,374,881 m^3^ to 25,481,486 m^3^), with a cumulative discharge >139 million m^3^, over the nine year period from 2005–2013 (Fig 3 and S2 Table). This increase was driven primarily by bulkers, which exhibited an even more rapid (1156%) increase from 2005–2013 and accounted for 76.2 ± 0.9% of total discharge per year in the last three years (Fig 3 and S2 Table). Overall, the annual number of overseas bulker arrivals was a strong predictor and explained most of the variation in total annual overseas BW discharge volume, when considering either discharge from all vessel types (R^2^ = 0.911, *p* <0.001) or bulkers alone (R^2^ = 0.916, *p* <0.001). In contrast to bulkers, the annual number of all overseas vessel arrivals (Fig 4) was a poor predictor of total overseas BW discharge volume (R^2^ = 0.02, *p* = 0.7153).

The observed temporal change in BW discharge was associated with coal exports (Fig 3). Specifically, annual overseas BW discharge volume was positively related to annual coal export quantity, whether considering total BW discharge from all vessel types or just that from bulkers (total discharge: R^2^ = 0.9923, *p* <0.001, bulker discharge: R^2^ = 0.9899, *p* <0.001).

These BW discharge data all correspond to the post-management era, when vessels arriving from overseas were required to manage BW in accordance with the USCG regulations. The estimated compliance rate of bulkers discharging overseas BW (coastal uptake location) into Chesapeake Bay ports was high: 99.1% of discharged BW was reported as having been exchanged, in compliance with existing regulations [45]. The initial BW uptake locations for nearly all bulkers travelling to Chesapeake Bay were in coastal waters (i.e. within 200 nmi of any shore), representing 96.3 ± 0.5% of annual BW discharged upon arrival. The remaining ~4% of BW was sourced in oceanic locations (i.e. > 200 nmi from any shore).

### Long-term changes in ship arrivals (1994–2013)

Total arrivals to Chesapeake Bay between 1994 and 2013 fluctuated between 4254 and 5333 vessels per year (Fig 4A). The majority of arrivals were coastwise. The annual number of bulker arrivals from overseas fluctuated greatly during this time period, representing between 5–15% of total arrivals (Fig 4B and 4C). Arrival numbers for overseas bulkers were lowest in 2001 and were three-fold higher in both 1995 and 2013 (Fig 4B). Between 2004 and 2013, annual arrivals of overseas bulkers to Chesapeake Bay ports increased 52% whereas that for total arrivals increased only 7%.

## Discussion

This study suggests that the abundance of zooplankton delivered annually from overseas BW to Chesapeake Bay has increased greatly in the past decade, during the transition from pre- to post-management era. This overall increase in propagule pressure was driven by two factors: (1) an increase of total zooplankton and coastal indicator zooplankton delivered in the post-management era and (2) an increase in total BW discharged in the post-management era. The NBIC data indicate a nearly 400% increase in annual BW discharge volume between 2005 and 2013, resulting from a remarkable (> 1,000%) increase in annual BW discharge volume by bulkers in the last three years (2011–2013), compared to 2005. Such large increases in BW discharge represent an unanticipated and unrecognised change, which has potential consequences for invasion dynamics and effectiveness of BW management.

Contrary to expectations, no reduction in zooplankton concentrations between the pre- and post-management eras was detected, despite the application of BWE reported by all vessels sampled in the post-management period (while no vessels used BWE in the pre-management era). Moreover, the observed concentrations of coastal indicator taxa, which would be expected to decline with the application of BWE, actually increased between pre- and post-management eras. Since cumulative zooplankton delivery per year results from the product of zooplankton concentration and BW volume, the data suggest that annual cumulative zooplankton delivery to Chesapeake Bay actually increased coincident with implementation of BWE (2001–2013), whether considering total zooplankton or coastal zooplankton. This increase in propagule supply is expected to increase the probability of establishment for new NIS or genotypes to a recipient region [15,24,48–50]. Below, the dynamics of each of these components are further explored and their implications for BW management are considered.

### Zooplankton concentrations over time

Upon initial evaluation, observed concentration of zooplankton in ballast water sampled in pre- versus post-management eras are contrary to expectations. In general, there is strong empirical and theoretical support that BWE reduces the original (coastal) biota in ballast tanks. The most reliable data on BWE efficacy result from controlled experiments that quantify changes (before vs. after BWE) in the concentration of target coastal organisms in a treated ballast tank, compared to an untreated tank on the same vessel [36,51]. This design controls for variation in initial concentrations, source region, voyage route, season, and ballast tank design that can affect survivorship and estimates for BWE effects on zooplankton. Some studies have also used this design to empirically measure changes in chemical tracer concentration, to evaluate efficacy of BWE to remove the original water volume. Results from such studies on commercial ships have consistently demonstrated high efficacy, resulting in 88–99% removal of target coastal organisms from the original water mass, though efficacy may vary by vessel type [29,51,52]. Estimates from these empirical studies are consistent with theoretical estimates of efficacy [e.g. 25,53,54] and indicate clearly that BWE reduces original zooplankton concentrations by roughly an order of magnitude, when performed properly (e.g. in approved BWE locations, flushing of 300% tank volume in flow through exchange).

There are several potential explanations that may account for relatively high concentrations in the post-management era samples. These higher concentrations may result from unexpected dynamics during one or more of the sequential stages in BW transfer between source and destination ports. First, it is possible that concentrations at initial uptake (prior to BWE) were higher in the post-management than the pre-management era, plausibly due to changing environmental factors or specific source locations [e.g. 55,56]. Assuming a high efficacy of BWE (~90% reduction of coastal organisms), this would require that initial zooplankton concentrations were an order of magnitude higher in post-management than pre-management era periods, due to temporal or spatial variation in the source zooplankton community. The major source ports differed in the pre- and post-management periods: European ports were dominant in the post-management era, while eastern Mediterranean ports were dominant in the pre-management era, and not represented at all in the post-management era. This analysis has attempted to control for gross temporal and spatial sources of variation, while examining ballast tanks for a single vessel type. For both eras, all samples were collected during the warm months of peak zooplankton abundance. The eastern Mediterranean is known to be oligotrophic, and the voyage duration to the U.S. from this region is relatively long. Both of these factors likely contribute to the low zooplankton concentrations observed for arrivals from this region (see Fig 2). Removing eastern Mediterranean source ports from the analyses, however, did not change the relative differences among eras for concentrations of total zooplankton or coastal indicator taxa. It certainly is possible that relatively high initial concentrations existed, either due to specific individual source ports or a decadal increase in zooplankton abundance, but without initial data at the point of origin or between eras this hypothesis cannot be tested.

Second, it is possible that survivorship during ocean transits somehow differed between eras, with much higher survivorship in the post-management era, allowing a higher proportion to arrive to Chesapeake Bay. In general, zooplankton survival in ballast tanks declines with voyage duration [27,28,34,39,40,57]; however, the source regions and voyage durations were similar for pre- and post-management eras (excluding the eastern Mediterranean), and removing the longer duration voyages did not alter the main results. Ballast water age was younger in the post-management era because the water was ‘refreshed’ during BWE, and this could explain why higher concentrations of zooplankton were observed in exchanged BW with voyage lengths up to 21 days (see Results). Increases in the abundance and diversity of plankton, though infrequent, have been observed after BWE [e.g. 29], and it has been suggested that such refreshing may sometimes have a rescue effect, allowing some organisms to persist longer in ballast tanks than would otherwise be the case. The evidence for this is largely circumstantial and has not been well documented for zooplankton [58,59]. In rarely reported cases, population increases have been observed due to reproduction during a voyage [39,60]. While reproduction may have contributed to total zooplankton abundance for some samples in this study, such effects would have been limited primarily to copepods, as most of the other groups are not expected generally to have adult source populations that can reproduce in ballast tanks. Moreover, given the short voyage durations, larval stages would not have sufficient time to mature and reproduce. Thus, removing larval and juvenile copepods from the analysis largely controls for any such in-transit reproduction, and did not alter the comparison of zooplankton concentrations between eras (Fig 1). Nonetheless, this study lacks repeated measures for the individual voyages, which would be needed to further evaluate any temporal differences in survivorship of zooplankton assemblages during transit.

A third possible explanation is that the efficacy of BWE was much lower than expected. All ships included in the post-management era reported BWE; these data are self-reported, however, and there are no independent data available to evaluate performance, including either validation that BWE occurred or that it was conducted properly. Some methods are available for such validation [61], but these are not applied routinely for arriving vessels. An analysis of vessels arriving to ports on the U.S. Pacific coast found discordance between reported BWE for some vessels and relatively high concentrations of chromophoric dissolved organic matter (CDOM), which is an effective tracer for residual coastal water, suggesting a subset of vessels did not properly perform BWE as reported [62]. Salinity can be used as an indicator that BWE has not been performed (i.e. if salinity <30 ppt); however, the high abundance of ports with marine waters often precludes the use of high salinity as confirmation that BWE has occurred [63]. As such, there is not sufficient data to evaluate BWE performance or efficacy for vessels arriving to Chesapeake Bay in the post-management era.

A fourth possibility is that differences in sampling and analysis methods somehow differed between eras, creating a systematic bias. Data presented in this study were collected over a 20-year period with multiple analysts processing samples, though identical methods were used to collect the samples. To limit the influence of analyst on the data presented all pre-management era samples were reanalysed, and subsequently compared to the current, post-management samples. A systematic bias was detected in the recounted pre-management samples, as reported in the Methods, whereby total zooplankton was 25.42% lower than initial counts (R^2^ = 0.9977). This may result from differences in analysts or degradation of preserved samples. The analyses presented in this study used the recounted data and adjusted for this temporal bias, to provide a conservative estimate of zooplankton concentrations in the pre-management era. If this 25% correction was not applied, post-management concentrations become even higher relative to the pre-management era. In short, this analysis does not support an analytical bias and suggests the temporal differences observed are robust.

The authors surmise that the relatively high concentrations of total zooplankton and coastal indicator taxa in the post-management era result from some combination of (1) increased initial concentrations, (2) enhanced survivorship, such as changing environmental conditions with BWE [e.g. 29] compared to historical samples, and (3) low performance of BWE as currently applied by vessels arriving to Chesapeake Bay. While the relative contribution of each potential mechanism is unknown, the net result is that zooplankton concentrations of overseas bulk vessels did not decline as predicted with the use of BWE. It is unlikely that BWE had no effect on a per vessel basis, since both empirical and theoretical evidence have demonstrated repeatedly that this treatment decreases zooplankton concentrations (when compared to control tanks on the same vessel), even when imperfectly applied. Instead, the authors hypothesise that the primary factor is a compensatory change that has occurred between pre- and post-management eras in the initial zooplankton concentrations of sampled vessels, leading to the observed net outcome by obscuring (counteracting) the per vessel effect of BWE. Such a change in initial concentrations could result simply from high variance in space and time [see 51], and the particular vessels (including source ports and routes) arriving to Chesapeake Bay in each era. Briski et al [13] also observed high variance and no clear effect of BWE on mean zooplankton concentrations, when comparing managed to unmanaged BW for coastwise arrivals entering Canadian ports from 2007–2009. This study extends that result to include such a comparison to overseas arrivals, contrasting pre- and post-management eras.

It is also important to recognise that our analyses do not adequately control for effects of time, port location, voyage route, or particular vessels in the comparison of zooplankton concentrations between eras, because each era has a unique combination of trade and shipping patterns, which change through time. These findings are not intended to be generalised to other time periods in Chesapeake Bay or other recipient regions, as voyage conditions that affect zooplankton communities may differ. The main results of this analysis are that BWE has not led to reduced net zooplankton propagule supply or a reduction in the proportion of coastal indicator taxa, in overseas BW between the historical (pre-management) and post-management eras in Chesapeake Bay. The extent to which this applies to other time horizons or geographic regions requires explicit evaluation, because of the dynamic nature of shipping and associated propagule supply in space and time. It does, however, highlight that datasets such as this (i.e. long-term datasets that control for time, port location, etc.) are vital to inform management decisions.

### Ballast water discharge volume over time

The massive, unexpected (374%) increase in annual overseas BW discharged into Chesapeake Bay identified by this study did not result from increasing total vessel traffic. Instead, an increase in bulker traffic, a result of growth in coal export from Chesapeake Bay, accounts for the observed increase in BW discharge from 2005–2013. The expansion in coal exports from Chesapeake Bay began in 2001 and was caused by global changes in demand, effects of weather events (e.g. in Australia and U.S.) on supply, changes in currency (exchange rates), transportation (fuel) costs and tariffs on coal [46]. These shifts in the coal and energy market are difficult to predict, being driven by diverse and complex forces.

From a BW delivery and management perspective, the increase in coal exports created a compensatory change in overseas propagule supply, acting in opposition to the effects of BWE, increasing cumulative annual delivery of total organisms and coastal organisms. Assuming a 90% reduction of coastal organisms’ concentrations due to BWE, only 10 of 100 initial coastal organisms would remain after BWE, but this becomes effectively 37.4 organisms when scaled to the increased volume. However, for zooplankton, the combination of increased BW volume and no reduction in organisms suggests that annual zooplankton (both total and coastal) concentrations delivered to Chesapeake Bay have increased between pre- and post-management eras. This study suggests that over 10 billion zooplankton were discharged in Chesapeake Bay (from overseas BW) in 2013 alone, likely representing the peak (to date) in total annual zooplankton delivery since the implementation of BWE. Specifically, the number of bulk vessel arrivals (1994–2013) exhibits a bimodal distribution, with comparable peaks in the mid-1990s to those observed in 2010–2013 (Fig 4). In addition, bulk vessel size (and hence BW volume per arrival) has likely increased with time. In short, such major shifts in shipping are a key aspect of evaluating BW management, because they can greatly affect the annual propagule supply and therefore invasion opportunity.

Looking to the future of Chesapeake Bay, further increases in shipping traffic could occur due to several major factors, including: 1) continued high overseas demand for coal, 2) the opening of the Panama Canal expansion and 3) the opening of a new offshore liquefied natural gas (LNG) shipping terminal. If demand for Chesapeake Bay coal exports continues, ballast water discharges will remain at the current high volume. The newly completed Panama Canal expansion may result in an influx of new generation Panamax-sized vessels to the U.S. Atlantic coast. At present, Norfolk and Baltimore are two of only a handful of ports on the U.S. Atlantic coast ready to receive these vessels. The commencement of this route could mean an increase in shipping traffic, the volume of BW discharged, and thus propagule pressure, to Chesapeake Bay [64]. Finally, there has been recent exploration of an LNG export facility in Maryland, which would further increase BW inputs to Chesapeake Bay by tankers [65]. All of these factors combined can lead to highly dynamic changes in BW delivery to Chesapeake Bay.

### Implications for BW management

Propagule pressure from BW to a particular geographic location is driven by multiple factors. These factors fluctuate through time, and can operate in a compensatory fashion, undermining current BW management strategies. This study demonstrates how fluctuations in trade dynamics can greatly increase total BW discharge volumes and also counteract expected effects of BWE on propagule concentrations. While BW discharge standards will replace BWE, implementation of treatment technologies that meet these standards has lagged. It is now 10 years since the adoption of the Ballast Water Management Convention, and systems are still in the development and certification stages. Recent ratification of the BWM Convention (September 2016) highlights the urgent need for these systems to be optimised, certified, and utilised by vessels to make significant advances to meet discharge standards for multiple organism types. The discharge standard for organisms’ that are ≥50 μm (dominantly zooplankton) is <10 live organisms per m^3^. This represents a reduction in zooplankton concentration of two orders of magnitude relative to post-management concentrations observed here for BW discharge in Chesapeake Bay (Fig 1). Equally important, the discharge standard is not prone to fluctuation (like BWE) due to initial source conditions or survivorship. Assuming compliance with the discharge standard, Chesapeake Bay would need to receive approximately 40 times the BW discharge (using 2013 data) to achieve similar propagule pressure. This is a massive increase, even from the current observed peak in Chesapeake Bay BW discharge levels. Clearly, this discharge standard should provide a significant reduction in propagule supply relative to BWE, and its use will remove a significant source of variation in the performance of current BW management, reducing one type of compensation that can undermine the efficacy of management efforts. This approach can mitigate changes observed in this study due to biological dynamics or BW discharge volume, providing a much needed advancement in BW management strategy.

## Supporting information

S1 Supporting Information This file includes the raw data and open access data source locations used to produce all figures in the manuscript. (DOCX)

S1 Table Zooplankton taxa designations. Designation of zooplankton into two categories: (1) coastal indicator taxa and (2) other non-indicator taxa.
(PDF)

S2 Table Temporal changes in ballast water discharge between 2005 and 2013. The volume of ballast water discharged between 2005 and 2013 for (1) overseas bulkers (coastal BW uptake location) and (2) total (all vessel types). The total annual percentage increase in BW discharge is shown for each.
(PDF)
