# Supplementary material for: Evaluating the combined effects of ballast water management and trade dynamics on transfers of marine organisms by ships
Source: PLoS One. 2017 Mar 20;12(3):e0172468. doi: 10.1371/journal.pone.0172468 (PMC5358743; doi:10.1371/journal.pone.0172468)
Supplement: S1 Supporting Information — (DOCX) [file pone.0172468.s001.docx]

Figures 1 and 2 data:

|  | **Zooplankton concentration data** | | |  |  |  |
| --- | --- | --- | --- | --- | --- | --- |
| **Voyage ID** | **Coastal indicator** | **Total** | **Total excluding copepod nauplii** | **Salinity** | **BW Age** | **Voyage length** |
| **Pre-management 1** | 4.641439 | 13.92432 | 13.92432 | 30 | 6 | 6 |
| **Pre-management 2** | 1.347343 | 8.757732 | 8.757732 | 40 | 16 | 16 |
| **Pre-management 3** | 379.876 | 1296.818 | 1231.322 | 26 | 10 | 10 |
| **Pre-management 4** | 148.5446 | 176.8388 | 176.8388 | 27 | 13 | 13 |
| **Pre-management 5** | 75.45123 | 730.9338 | 400.8347 | 36 | 14 | 14 |
| **Pre-management 6** | 9.431404 | 80.16693 | 70.73553 | 30 | 34 | 34 |
| **Pre-management 7** | 1081.243 | 1152.652 | 1122.337 | 28 | 13 | 13 |
| **Pre-management 8** | 21.5282 | 743.6457 | 385.0473 | 32.25 | 11 | 11 |
| **Pre-management 9** | 38.02985 | 2307.145 | 2294.468 | 23.1 | 15 | 15 |
| **Pre-management 10** | 101.6823 | 384.6244 | 309.4679 | 29.35 | 14 | 14 |
| **Pre-management 11** | 397.8874 | 1304.186 | 806.8271 | 33.75 | 11 | 11 |
| **Pre-management 12** | 259.3636 | 613.0413 | 514.0115 | NA | 6 | 6 |
| **Pre-management 13** | 11.78926 | 23.57851 | 17.68388 | 17 | 21 | 21 |
| **Pre-management 14** | 1779.106 | 5176.555 | 3150.946 | 32 | 12 | 12 |
| **Pre-management 15** | 0 | 954.9297 | 0 | 8.25 | 11 | 11 |
| **Pre-management 16** | 14.14711 | 51.87272 | 14.14711 | 37.5 | 22 | 22 |
| **Pre-management 17** | 25.46479 | 70.73553 | 67.90611 | 36.65 | 16 | 16 |
| **Pre-management 18** | 0 | 18.25433 | 13.69075 | 32 | NA | NA |
| **Pre-management 19** | 15.71901 | 78.59503 | 69.16363 | 5 | 13 | 13 |
| **Pre-management 20** | 9.226374 | 31.98476 | 21.5282 | 42 | 20 | 20 |
| **Pre-management 21** | 9.431404 | 74.77756 | 54.56741 | 38.25 | 17 | 17 |
| **Pre-management 22** | 224.5366 | 401.051 | 306.3043 | 38 | 17 | 17 |
| **Pre-management 23** | 8.630043 | 20.95868 | 14.17793 | 4.5 | 14 | 14 |
| **Pre-management 24** | 2.122066 | 2.829421 | 2.829421 | 25 | 18 | 18 |
| **Pre-management 25** | 69.16363 | 657.0545 | 638.1917 | 25 | 23 | 23 |
| **Pre-management 26** | 5460.783 | 64906.92 | 14118.81 | NA | NA | 8 |
| **Pre-management 27** | 21.22066 | 56.58842 | 49.51487 | 36.47 | 19 | 19 |
| **Pre-management 28** | 40.88384 | 83.71453 | 72.68238 | 31.8 | 16 | 16 |
| **Pre-management 29** | 49.90353 | 533.0039 | 154.9963 | 6.85 | 12 | 12 |
| **Pre-management 30** | 0 | 17671.47 | 5400.72 | 4.4 | 12 | 12 |
| **Pre-management 31** | 622.4727 | 5998.373 | 5828.608 | 17.2 | 12 | 12 |
| **Pre-management 32** | 1.806014 | 9.030068 | 7.826059 | 40.5 | 22 | 22 |
| **Pre-management 33** | 124.2185 | 820.0721 | 376.106 | 40 | 10 | 10 |
| **Pre-management 34** | 11.31768 | 141.4711 | 113.1768 | NA | 6 | 6 |
| **Pre-management 35** | 22.46893 | 87.37918 | 59.91716 | NA | 2 | 2 |
| **Post-management 1** | 1547.071 | 2492.503 | 2005.463 | 33.4 | 6 | 14 |
| **Post-management 2** | 353.7918 | 2907.94 | 1716.461 | 35.5 | 3 | 5 |
| **Post-management 3** | 3059.67 | 8864.738 | 7974.845 | 22.7 | 7 | 16 |
| **Post-management 4** | 51.24149 | 84.65985 | 69.06461 | 37.1 | 7 | 18 |
| **Post-management 5** | 133.1155 | 237.56 | 202.7452 | 34.1 | 6 | 8 |
| **Post-management 6** | 51.60039 | 303.1523 | 266.602 | 33.7 | 11 | 18 |
| **Post-management 7** | 158.245 | 803.8848 | 468.4053 | 36.8 | 4 | 18 |
| **Post-management 8** | 428.6268 | 467.3727 | 431.0484 | 37 | 13 | 25 |
| **Post-management 9** | 729.1731 | 2882.243 | 1524.374 | 37.1 | 9 | 13 |
| **Post-management 10** | 3.762528 | 26.3377 | 26.3377 | 36.3 | 7 | 14 |
| **Post-management 11** | 6.477613 | 6.477613 | 6.477613 | 36.8 | 11 | 24 |
| **Post-management 12** | 63.39932 | 3214.808 | 2374.767 | 36.6 | 9 | 15 |
| **Post-management 13** | 33.52395 | 2457.563 | 1472.475 | 31 | 2 | 6 |
| **Post-management 14** | 241.3548 | 5592.934 | 5196.091 | 37.1 | 7 | 13 |
| **Post-management 15** | 246.1222 | 7770.431 | 1406.413 | 35.2 | 11 | 16 |
| **Post-management 16** | 190.2888 | 4219.798 | 555.2973 | 36.4 | 20 | 29 |
| **Post-management 17** | 94.0665 | 2830.659 | 2219.227 | 37 | 14 | 19 |
| **Post-management 18** | 551.7719 | 11972.64 | 9189.436 | 35.6 | 13 | 18 |
| **Post-management 19** | 1116.073 | 34950.71 | 12981.69 | 35.1 | 2 | 3 |
| **Post-management 20** | 18.41472 | 66.75335 | 46.03679 | 35.9 | 18 | 33 |
| **Post-management 21** | 1740.111 | 16168.54 | 11238.22 | 33.7 | 6 | 15 |
| **Post-management 22** | 0 | 10412.61 | 1871.929 | 35.7 | 10 | 17 |
| **Post-management 23** | 45.65055 | 214.5576 | 143.7992 | 35.9 | 4 | 9 |
| **Post-management 24** | 1203.336 | 12342.79 | 5810.394 | 35.4 | 13 | 21 |
| **Post-management 25** | 118.1269 | 2179.723 | 680.6361 | 35.8 | 10 | 15 |
| **Post-management 26** | 1074.298 | 4853.025 | 2749.269 | 31 | 8 | 14 |
| **Post-management 27** | 9.668607 | 59.94536 | 15.46977 | 36.6 | 26 | 38 |
| **Post-management 28** | 627.3751 | 3744.013 | 1821.412 | 33.2 | 8 | 17 |
| **Post-management 29** | 381.5293 | 6867.527 | 4101.44 | 30.9 | 1 | 6 |
| **Post-management 30** | 93.82976 | 696.1563 | 441.9079 | 35.2 | 9 | 15 |
| **Post-management 31** | 26.52582 | 28.73631 | 28.73631 | 35.1 | 12 | 18 |
| **Post-management 32** | 1921.219 | 5344.745 | 4304.686 | 36.3 | 10 | 17 |
| **Post-management 33** | 37.40422 | 739.7723 | 249.3614 | 35.6 | 9 | 14 |
| **Post-management 34** | 88.604 | 240.4966 | 191.9753 | 35.8 | 10 | 14 |
| **Post-management 35** | 197.1353 | 235.2905 | 199.255 | 34.6 | 13 | 21 |
| **Post-management 36** | 44.92095 | 44.92095 | 44.92095 | 35.2 | 14 | 21 |
| **Post-management 37** | 2.210485 | 4.420971 | 4.420971 | 37 | 9 | 23 |

Figure 3 data:

Coal data available from the EIA coal data browser: <http://www.eia.gov/beta/coal/data/browser/>

Ballast water discharge data is available on the National Ballast Information Clearinghouse website: <http://invasions.si.edu/nbic/>

Figure 4 data:

| **Year** | **Total arrivals: Marad** | **Total arrivals: NVMC** | **Overseas Bulker arrivals: Marad** | **Overseas Bulker arrivals: NVMC** | **Bulker % of total: Marad** | **Bulker % of total: NVMC** |
| --- | --- | --- | --- | --- | --- | --- |
| **1994** | 4886 | #N/A | #N/A | #N/A | #N/A | #N/A |
| **1995** | 5023 | #N/A | 669 | #N/A | 13.31873382 | #N/A |
| **1996** | 5025 | #N/A | 738 | #N/A | 14.68656716 | #N/A |
| **1997** | 4720 | #N/A | 576 | #N/A | 12.20338983 | #N/A |
| **1998** | 4486 | #N/A | 563 | #N/A | 12.55015604 | #N/A |
| **1999** | 4354 | #N/A | 425 | #N/A | 9.761139182 | #N/A |
| **2000** | 4324 | #N/A | 422 | #N/A | 9.759481961 | #N/A |
| **2001** | 4644 | #N/A | 228 | #N/A | 4.909560724 | #N/A |
| **2002** | 4343 | #N/A | 306 | #N/A | 7.045820861 | #N/A |
| **2003** | 4513 | #N/A | 303 | #N/A | 6.713937514 | #N/A |
| **2004** | 4779 | 4661 | 456 | 500 | 9.541745135 | 10.7273117 |
| **2005** | 4848 | 4544 | 378 | 422 | 7.797029703 | 9.28697183 |
| **2006** | #N/A | 5133 | #N/A | 333 | #N/A | 6.48743425 |
| **2007** | #N/A | 5333 | #N/A | 411 | #N/A | 7.70673167 |
| **2008** | #N/A | 5237 | #N/A | 619 | #N/A | 11.8197441 |
| **2009** | #N/A | 4608 | #N/A | 519 | #N/A | 11.2630208 |
| **2010** | #N/A | 4924 | #N/A | 626 | #N/A | 12.7132413 |
| **2011** | #N/A | 4973 | #N/A | 731 | #N/A | 14.6993766 |
| **2012** | #N/A | 5038 | #N/A | 775 | #N/A | 15.3830885 |
| **2013** | #N/A | 5027 | #N/A | 760 | #N/A | 15.1183609 |
