# Supplementary material for: Evaluating the combined effects of ballast water management and trade dynamics on transfers of marine organisms by ships
Source: PLoS One. 2017 Mar 20;12(3):e0172468. doi: 10.1371/journal.pone.0172468 (PMC5358743; doi:10.1371/journal.pone.0172468)
Supplement: S1 Table — Designation of zooplankton into two categories: (1) coastal indicator taxa and (2) other non-indicator taxa. (PDF) [file pone.0172468.s002.pdf]

S1 Table. Zooplankton taxa designations.

Taxon	Common name	Coastal indicator taxa	Other non-indicator taxa
Crustacea: Malacostraca: amphipoda	Amphipod	X	
Chordata: Appendicularia	Appendicularian		X
Crustacea: Cirripedia	Barnacle Larva	X	
Mollusca: Bivalvia	Bivalve	X	
Bryozoa	Bryozoan Larva	X	
Crustacea: Copepoda: Calanoida	Calanoid		X
Crustacea: Malacostraca: Amphipoda	Caprellid	X	
Chaetognatha: Sagittoidea	Chaetognatha		X
Crustacea: Branchiopoda: Cladocera	Cladoceran	X	
Crustacea: Copepoda	Copepod Adult		X
Crustacea: Copepoda	Copepod Nauplius		X
Crustacea: Malacostraca: Decapoda	Crab	X	
Crustacea	Crustacean Larva	X	
Ctenophora	Ctenophore		X
Crustacea: Copepoda: Cyclopoida	Cyclopoid		X
Echinodermata	Echinoderm Larva	X	
Crustacea: Malacostraca: Euphausiacea	Euphausid	X	
Platyhelminthes	Flatworm	X	
Foraminifera	Foram	X	
Mollusca: Gastropoda	Gastropod	X	
Crustacea: Copepoda: Harpacticoida	Harpacticoid	X	
Crustacea: Malacostraca: Isopoda	Isopod	X	
Cnidaria: Medusozoa	Medusa	X	
Arthropoda: Arachnida	Mite	X	
Platyhelminthes	Mullers Larva	X	
Crustacea: Malacostraca: Mysida	Mysid	X	
Nematoda	Nematode	X	
Crustacea: Ostrocoda	Ostrocod	X	
Crustacea: Copepoda: Poecilostomatoida	Poecilostom		X
Annelida: Polychaeta	Polychaete Adult	X	
Annelida: Polychaeta	Polychaete Larva	X	
Radiolaria	Radiolarian		X
Rotifera	Rotifer	X	
Chordata: Thaliacea: Salpida	Salp		X
Crustacea	Shrimp	X	
Annelida: Polychaeta: Canalipalpata	Spionid	X	
Crustacea: Malacostraca: Stomatopoda	Stomatopod	X	
Ciliophora: Spirotrichea: Tintinnida	Tintinnid		X
Multiple	Trochophore	X	
