# Supplementary material for: Evaluating the combined effects of ballast water management and trade dynamics on transfers of marine organisms by ships
Source: PLoS One. 2017 Mar 20;12(3):e0172468. doi: 10.1371/journal.pone.0172468 (PMC5358743; doi:10.1371/journal.pone.0172468)
Supplement: S2 Table — The volume of ballast water discharged between 2005 and 2013 for (1) overseas bulkers (coastal BW uptake location) and (2) total (all vessel types). The total annual percentage increase in BW discharge is shown for each. (PDF) [file pone.0172468.s003.pdf]

S2 Table. Temporal changes in ballast water discharge between 2005 and 2013.

Year	Overseas bulker BW discharge (coastal uptake location) (MT)	Increase in overseas bulker BW discharge since 2004	Total BW discharge (MT)	Increase in total BW discharge since 2004	Overseas bulker BW discharge as percentage of total BW discharge
**2005**	1,569,007	0%	5,374,882	0%	29%
**2006**	1,916,846	22%	6,393,620	19%	30%
**2007**	4,002,957	155%	8,608,043	60%	47%
**2008**	8,147,410	418%	13,307,036	148%	61%
**2009**	7,603,792	383%	11,556,563	115%	66%
**2010**	11,581,301	638%	16,717,067	211%	69%
**2011**	18,001,060	1022%	24,194,375	350%	74%
**2012**	21,267,764	1222%	27,649,381	414%	77%
**2013**	19,704,205	1156%	25,481,486	374%	77%
